# The neurological assessment in young children treated with artesunate monotherapy or artesunate-mefloquine combination therapy for uncomplicated *Plasmodium falciparum *malaria

**DOI:** 10.1186/1475-2875-8-207

**Published:** 2009-09-02

**Authors:** Michael T Ambler, Lilly M Dubowitz, Ratree Arunjerdja, Eh Paw Hla, Kyaw Lay Thwai, Jacher Viladpainguen, Pratap Singhasivanon, Christine Luxemburger, François Nosten, Rose McGready

**Affiliations:** 1 Shoklo Malaria Research Unit, PO Box 46, Mae Sot, Tak, Thailand, 63110; 2 Department of Paediatrics, Imperial College, Hammersmith Hospital, Du Cane Rd, London W12 OHS, UK; 3 Mahidol-Oxford Tropical Medicine Research Unit (MORU), Faculty of Tropical Medicine, Mahidol University, Bangkok, Thailand; 4 Centre for Tropical Medicine, Nuffield Department of Clinical Medicine, University of Oxford, CCVTM, Oxford OX3 7LJ, UK

## Abstract

**Background:**

Mefloquine and artesunate combination therapy is the recommended first-line treatment for uncomplicated malaria throughout much of south-east Asia. Concerns have been raised about the potential central nervous system (CNS) effects of both drug components and there are no detailed reports in very young children.

**Methods:**

Children, aged between three months and five years, with acute uncomplicated *Plasmodium falciparum *malaria were randomized to either 7 days of artesunate monotherapy or the same schedule of artesunate plus mefloquine on day 7 and 8. Neurological testing targeting coordination and behaviour was carried out at day 0, 7, 9, 10, 14 and 28. Non-febrile healthy control children from the same population were tested on days 0, 7, 14 and 28.

**Results:**

From December 1994 to July 1997, 91 children with uncomplicated *P. falciparum*, 45 treated with artesunate monotherapy, 46 treated with mefloquine and artesunate combination therapy and 36 non-febrile controls, underwent neurological testing. Malaria and fever had a significant negative impact on testing performance. By contrast, the anti-malarial treatments were not associated with worsening performances in the various components of the test. Artesunate and mefloquine do not appear to have a significant influence on coordination and behaviour. Children treated with mefloquine were significantly less likely to suffer recurrent malaria infection during follow-up compared to those treated with artesunate alone (P = 0.033).

**Conclusion:**

In keeping with the results of randomized controlled trials in adults, mefloquine was not associated with a decrease in specific items of neurological performance. Likewise, children treated with artesunate did not perform significantly differently to control children. This study does not exclude subtle or rare treatment CNS effects of artesunate or mefloquine. Treatment of acute uncomplicated malaria results in a significant improvement on items of neurological performance.

## Background

In 2006, the WHO recommended the use of artemisinin combination therapy (ACT) as the first-line treatment of uncomplicated falciparum malaria[1]. Mefloquine in combination with artesunate has been the first-line treatment on the western border of Thailand for 15 years and is recommended in much of south-east Asia [2], where strains of multiple-drug-resistant parasites are common [3]. The safety and efficacy of mefloquine-artesunate combination treatment for uncomplicated falciparum malaria is well-established [4-7].

Since the study reported here was undertaken (in 1994), the CNS adverse effects and particularly the neuropsychiatric effects of mefloquine have been the focus of publications including several reviews [8-11]. Published data on the neuropsychiatric effects of mefloquine come from more than 25,000 patients (20 trials) with dizziness and anxiety reported most commonly in adults [11,12], as well as vomiting, especially in young children [9]. These effects are dose-related [8]. There were concerns that the CNS adverse effects reported by the manufacturer such as dizziness, vertigo and headache could affect those operating machinery and this was studied in adults. There was no functional compromise in American soldiers who took weekly mefloquine (preceded by the loading dose) as prophylaxis, despite more reporting sleep disturbances, increased dream activity and depression [13]. In a double-blind, placebo-controlled, cross-over study of 23 Swiss trainee pilots, mefloquine was given as a loading dose, followed by weekly administration for three weeks [14]. There were no significant differences in flying performance, psychomotor functions and postural sway between the two arms, although a non-significant reduction in sleep quality was reported by the mefloquine recipients. Similar findings were reported in another placebo-controlled trial of mefloquine prophylaxis and the effect of alcohol when driving a car [15]. In 10 volunteers, mefloquine had no effect on audiometry and vestibular functions [16]. A study of 73 volunteer Dutch marines, stationed in Cambodia, who used mefloquine for prophylaxis also reported a low risk of adverse events, including coordination problems, during the three-month follow up [17]. These data suggest that, although mefloquine is often associated with CNS effects, it does not interfere with coordination tasks requiring higher cognitive function in adults. Data on the neuropsychiatric effects of mefloquine in young children is limited to a single study with mefloquine monotherapy, which found no evidence for such an effect in children less than five years of age, when treated for *falciparum *or *vivax *malaria[18]. As described by the authors, they lacked a sensitive and objective tool for the assessment of neurological status in this age group and any neurological disturbance less catastrophic than convulsion, major behavioural change, or encephalopathy could not be excluded.

The artemisinin derivatives, characterized by Chinese scientists in the early 1970s [19-21], have been associated with CNS toxicity in animals. In one study, dogs treated with high doses of intramuscular artemether or arteether developed gait disturbances, loss of spinal and pain response reflexes, and prominent loss of brain stem and eye reflexes. This lead to cardiorespiratory depression and death in five out of six animals, as a result of selective damage to the brain stem, particularly to the reticular formation, the vestibular system and nuclei related to the auditory system [22]. Rats and Rhesus monkey given arteether or artemether showed a similar selective pattern of brain stem pathology [23,24]. These neurotoxic effects have not been found in human trials that have examined coordination (heel-toe ataxia), fine finger dexterity, hearing, nystagmus and balance (Romberg's test) in patients (>5 years) treated with artemether or artesunate as monotherapy or with mefloquine [25]. Selected testing for audiometry and auditory evoked potentials in patients treated with artemisinin derivatives [26,27] or with the combination of artesunate and mefloquine [28] also failed to find any detrimental artemisinin effect. In addition, brainstems of adults who died after treatment with high dose artemether or quinine for severe falciparum malaria showed no evidence of selective neuronal damage[29].

This aim of this study was to examine, in a resource-poor setting, the potential neurotoxicity of treatment doses of artesunate and mefloquine given to young children (< 5 years age) with acute falciparum malaria. No single standardized test that focused on ataxia, irritability, behavioural and tone change in children of this age, and could be easily applied in remote clinics by local health workers, existed for this problem. Hence items of interest that specifically examined for coordination and behaviour were selected from previously standardized tests. Age-matched healthy children from the same community were included as controls to elucidate practice effects and as a comparator group for normal neurological test results in this age group.

## Methods

This study was approved by the ethical committee of the Faculty of Tropical Medicine, Mahidol University, Bangkok and the Karen Ethics Committee, Mae Sot, Thailand.

### Participants

Young children from three months to five years of age, attending the clinics of Shoklo Malaria Research Unit in Maela Refugee camp on the western border of Thailand with Burma, were considered for enrolment if they presented with symptomatic, microscopically confirmed *Plasmodium falciparum *malaria. They were excluded if they had signs of severe or complicated malaria[30], a concomitant severe disease which required hospitalization, or any underlying neuro-developmental condition, including epilepsy. Controls were not randomly selected. They were the relatives of children in the study who were selected because their parents volunteered them after invitation, they had a negative malaria smear, and were free from medications and fever for at least 48 hours, and had a normal body temperature prior to neurological testing.

### Protocol

If consent was forthcoming a full medical history and clinical examination was carried out. This included assessing the duration of symptoms before presentation and any drugs taken prior to arrival at the clinic. Daily malaria smears were made until the patient was malaria smear negative. Temperature was measured daily until the patient was afebrile. Patients under treatment had daily clinical examination, drug administration, and adverse events documented on a case record form.

### Anti-malarial drug treatments

Participants with acute uncomplicated falciparum malaria received either artesunate alone (AS7), or artesunate in combination with mefloquine (MAS7). The total dose of artesunate was 12 mg/kg and the total dose of mefloquine was 25 mg/kg. Treatment regimes were as follows:

AS7: Artesunate 2 mg/kg for days 0 to 4, then 1 mg/kg for days 5 and 6.

MAS7: Artesunate 2 mg/kg for days 0 to 4, then 1 mg/kg on days 5 and 6; Mefloquine 15 mg/kg day 7 and 10 mg/kg day 8.

The protocol for drug administration was to calculate the target dose per body weight, crush the tablets in water and give with sugar, biscuits or milk. This was done by injecting the suspension into the mouth with a 5-ml syringe followed by milk and food if tolerated. If vomiting occurred within the first 30 minutes, the entire dose was repeated. If vomiting occurred between 30 minutes and 1 hour later, half the initial dose was repeated. Repeat dosing was given via nasogastric tube.

Reappearance of *P. falciparum *was treated with 7 days of artesunate, and mefloquine if it had not been prescribed in the previous 2 months. Treatment of *Plasmodium vivax *infections was with a standard chloroquine regimen (25 mg base/kg over 3 days).

### Clinical outcome

Clinical assessment was performed daily from day 0 to day 5, then on days 7, 9, 10, then weekly until week 9. Only the results up to day 28 are reported here. Follow-up included basic clinical examination and weekly malaria smear and haematocrit from day 0. In addition, on each follow-up day, the parents or guardians of the participant completed a questionnaire regarding occurrence of symptoms and side-effects.

### Neurological outcome

Children with malaria were tested on days 0, 7, 9, 10, 14 and 28. Children completed the neurological assessment before treatment on day 0, and on all other days were treated then assessed. Control children were invited for neurological examination on days 0, 7, 14 and 28. Testing until day 28 was considered necessary due to the pharmacokinetic properties of mefloquine[31]. Mefloquine was administered on days 7 and 8 so both treatment groups had repeat neurological testing on days 9 and 10.

Neurological items predominantly assessing hand coordination (box, cube, ring, coin, and a timed item to place 6 and 12 coins into a slot in a coin box as fast as possible) were adapted from the Griffiths Developmental Scales [32] and the Movement ABC [33]; those assessing tone and behaviour from the Hammersmith Infant Neurological Examination [34]and the Bayley Scales of Infant Development[35]. The complete testing procedure, applicability and scoring in this population have been described previously [36]. These tests were chosen because they focused on coordination and concentration which were the CNS adverse affects of mefloquine and artesunate that were of greatest concern at the time. While an adult can be asked about dizziness young children cannot but it may be observed in items testing coordination by for example ataxic movements.

### Randomization

Children were randomized to one of two treatment groups using a list of random numbers that were allocated in a 1:1 ratio in blocks of 10. The randomization list was generated by an independent statistician who was not involved in conducting the study. The treatment allocation was concealed in envelopes labeled with the study code by another person not involved in conducting the study. The study envelopes were sorted by code and kept at the field sites. Inclusion was sequential. Patients who met the inclusion criteria were assigned the next available code. The envelope was then opened and the patient treated according to the allocation.

### Blinding

The testing room was separate to the medical outpatient department. Testers were kept blind to the treatment group of the patients with malaria. Testers knew only when patients belonged to the control group.

### Laboratory procedure

Blood smears (thin and thick films) were prepared using Giemsa staining and were read for 200 fields before being declared negative. Uncomplicated falciparum malaria was defined as slide-confirmed *P. falciparum*, with an asexual parasitaemia (between 6/500 white blood cells and 40/1,000 red blood cells, equivalent to 96 - 150,000 parasites/uL), in the absence of signs of severe malaria. Hyperparasitaemia was defined by a parasitaemia of ≥ 40/1000 RBC.

Haematocrit capillary tube samples were taken by finger prick and centrifuged at 1,500 rev/min for three minutes using a standard Hawksley™ haemotocrit reader. Anaemia was defined as a haematocrit of less than 30%, and severe anaemia by a haematocrit <20%.

### Sample size calculation

The combination of mefloquine and artesunate was expected to cause a 3 fold increase in worse outcomes based on the increase in severe dizziness found in children treated with high dose mefloquine in this area [37]. A sample size of 42 children in each group allowed a 3 fold higher incidence of worse outcomes (15 to 45%), to be detected with 95% confidence and 80% power. A 15% drop out was expected and 6 was added i.e 49 in each group.

### Statistical methods

Only children who completed a full course of treatment according to the protocol and who had at least two days of neurological testing, which included a test on day 0 (baseline), were included. Rather than use the overall scores for the items of hand co-ordination, behaviour and tone, some of which were age dependent, results of testing on a specified day were compared to a prior day e.g. day 7 compared to day 0. Children could refuse to participate in tests although they were all of an age where they could potentially score on the test item. Hence a child might refuse one week and get a top score the next week or vice versa. Therefore for each item examined we chose to assign the score as performed worse (score = 1), performed the same (score = 2) or improved performance (score = 3) in comparison to the previous score. Behaviour was scored in the same way and tone was scored as tone decreased (score = 1), tone stayed the same (score = 2) or tone increased (score = 3). Neurological test results could no longer be used once children were diagnosed with recurrent malaria of any species. Differences in proportions were compared using the Chi-squared test or Fisher's exact test when appropriate. Normally distributed data was compared using the mean and Student's T test. Data that was not normally distributed was summarized by the median and compared using the Mann Whitney U test when independent or with Wilcoxon Signed Ranks Test when paired at specified days. Statistical programmes used were SPSS for Windows, version 11.0 (SPSS), and Epi Info, version 3.4.0 (Centres for Disease Control and Prevention).

## Results

### Study participants

Ninety-eight patients between the ages of three months and five years with uncomplicated falciparum malaria were randomized between 5^th ^December 1994 and 29^th ^July 1997. Six patients did not have any paired neurological data and one patient was treated before testing on day 0 and all these children were excluded from analysis. Of the remaining 91 children, 45 were treated with artesunate alone and 46 were treated with mefloquine and artesunate. There were 36 control children, with a mean age of 2.2 ± 1.2 [0.3-4.7] years, of whom 61.1% (22/36) were male which was not significantly different from the children with malaria (P = 0.871, P = 0.574, respectively).

In children with malaria, there were no significant differences between treatment groups on admission characteristics (Table 1) or symptoms on admission (Table 2). After starting treatment fever and other symptoms decreased, and there was no significant difference in the proportion of children who developed new symptoms (data not shown). There was no difference in fever clearance time between the groups. Participants who became smear-positive for either *P. falciparum *or *P. vivax *during follow-up were retreated. Significantly more participants in the AS7 group become positive for malaria by day 28 than in the MAS7 group: 21.7% (10/46) *vs *4.4% (2/45), P = 0.033. In the AS7 group one child had a mixed infection (*P. falciparum *and *P. vivax*) identified by day 14; the remaining nine all had *P. vivax*, one of whom was diagnosed by day 14, and the rest were diagnosed by day 28. In the MAS7 group two children had *P. falciparum *identified at day 28.

**Table 1:** Clinical characteristics on admission in the AS7 and MAS7 groups

**Characteristic**	**AS7 n = 45**	**MAS7 n = 46**	**P**
Age, yrs, mean	2.3 ± 1.3 [0.4-5.0]	2.3 ± 1.3 [0.3-4.7]	0.940
Male, %	57.8 (26/45)	50.0 (23/46)	0.530
Weight, kg, mean	10 ± 3 [5-15]	10 ± 3 [5-17]	0.893
Height, cm, mean	80 ± 11 [61-102]	81 ± 10 [56-106]	0.891
PR, bpm, mean	130 ± 15 [100-160]	132 ± 14 [110-160]	0.645
Temperature, °C, mean	37.8 ± 1.0 [36-40.2]	37.9 ± 1.0 [36-40.0]	0.483
Proportion febrile, %	55.6 (25/45)	56.5 (26/46)	1.000
Palpable spleen, %	45.5 (20/44)	38.6 (17/44)	0.666
Palpable liver, %	52.3 (23/44)	47.7 (21/44)	0.831
Early vomiting day0, %	17.8 (8/45)	28.3 (13/46)	0.321
Haematocrit, %	30.3 ± 5.2 [16.7-39.5]	29.2 ± 5.5 [15.2-39.8]	0.329
Geometric mean parasitaemia/uL	3,981 [145-109,648]	3,090 [16-97,724]	0.575
History fever, days, median	2 [1-10]	2 [0-10]	0.925

Data % (n) unless otherwise stated;

Proportions compared with Chi-square test (or Fisher' exact), means with Student's t-test and medians with Mann Whitney U test.

**Table 2:** Symptoms on admission in the AS7 and MAS7 groups

**Symptoms**	**AS7 n = 45**	**MAS7 n = 46**	**P**
Feels ill	97.8 (45/45)	97.8 (44/46)	1.000
Weakness	68.2 (30/44)	65.2 (30/46)	0.825
Stopped walking, sitting or crawling	42.2 (19/45)	34.8 (16/46)	0.522
Talk/babbles less	40.0 (18/45)	43.5 (20/46)	0.832
Feeding less	70.5 (31/44)	68.9 (31/45)	1.000
Refuse food	60.5 (26/43)	50.0 (22/44)	0.391
Sleeping badly	57.8 (26/45)	45.7 (21/46)	0.297
Crying more than normal	71.71 (32/45)	71.7 (33/46)	1.000
Playing less than normal	86.7 (39/45)	84.8 (39/46)	1.000
Eye contact reduced	13.6 (6/44)	21.7 (10/46)	0.411
Seek mum/guardian	48.9 (22/456)	39.1 (18/46)	0.402
Sad/depressed	75.6 (34/456)	76.1 (35/46)	1.000
Less responsive to mum	48.9 (22/45)	54.3 (24/46)	0.677
Strange behaviour	4.3 (2/45)	4.3 (2/46)	1.000
Smiling less	84.4 (38/45)	82.6 (38/46)	1.000

### Valid test scores

**Figure 1:**
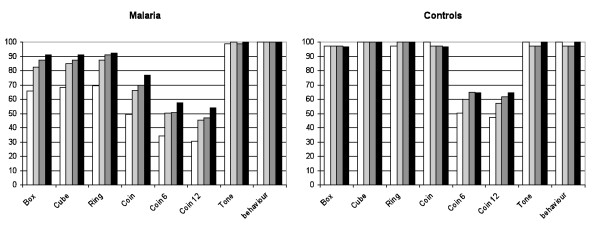
**Proportion of neurological items with a valid test score in young children, malaria and controls, at each day of follow-up, Day 0 (white square), Day 7 (light grey square), Day 14 (dark grey square) and day 28 (black square)**.

Many children refused testing on one or more items. The proportion of valid test scores was consistently lower in the malarial children than control children from day 0 to day 28 (Figure 1). The differences between malaria and control children were significant for box, cube, ring and coin items on day 0 (P < 0.001 for all) and on day 7 (P = 0.037, P = 0.019, P = 0.032, P < 0.001, respectively), day 14 cube (P = 0.037) and coin (P = 0.001) and on day 28 only coin (P = 0.012). As the days post-treatment increased there was a tendency in the malaria group towards increasing proportions of valid test scores however it never reached the same high proportion as the control children. By day 28, only the coin task showed significantly fewer valid test scores in the malaria group compared to the control group (Figure 1). There were no significant differences between the AS and MAS7 treatment groups in the proportion of valid test scores (data not shown) at each day of follow-up, on any neurological item. Tone and behaviour test scores were nearly always valid (Figure 1) regardless of the day of testing or group.

On day 0 children with fever were less likely to have valid test scores than non-febrile children for most items: box 62.7% (32/51) *vs *82.9 (63/76), P = 0.013; cube 62.7% (32/51) *vs *86.8% (66/76), P = 0.002; and ring 64.7% (33/51) *vs *85.5% (65/76), P = 0.009. The proportion of febrile children who were timed while putting 6 or12 coins through a slot in a box (coin 6 and 12) was less than for non-febrile children but the difference was not significant. The median tone score in children with fever compared to those without fever was significantly higher 11 [8-15]*vs *11 [6-15], P = 0.020. The median behaviour score in children with fever compared to those without fever was significantly lower: 11 [6-15]*vs *14 [6-15], P < 0.001. Tone was negatively and significantly correlated with behaviour (P = 0.007). Vomiting was also associated with a significant increase in tone and decrease in behaviour scores compared to those who did not vomit: 12 [8-15]*vs *11 [6-15], P = 0.013; 10 [6-15]*vs *13 [6-15], P = 0.042, respectively.

### Analysis of performance on day 7 and 28 compared to day 0

Up to day 7, both malaria treatment groups received identical treatment with artesunate alone. There were no significant differences in the proportion of hand co-ordination, behaviour or tone scores that were worse, compared to the same or improved, in the AS7 *vs *MAS7 groups for any item (Table 3). There were also no differences between malaria and controls on items of hand co-ordination on the day 7 - day 0 comparison or on the day 28 - day 0 comparison, except for the ring task, which was significantly worse in the controls. Behaviour improved significantly more in the malaria treated children compared to controls at both day 7 to day 0 and day 28 to day 0, with most of the improvement apparent by day 7. Concomitant to the fever clearance, tone decreased significantly in malaria treated children at both the day 7 to day 0 and day 28 to day 0 comparisons (Table 3).

**Table 3:** Proportion of children with worse performance on day 7 and day 28 compared to day 0, after treatment with AS7 or MAS7 for *P. falciparum*, and in the non febrile controls.

		**Day 7 - Day 0**			
**Neurological Test** **Component**	**Result compared to previous test**	**AS7** **N = 39**	**MAS7** **N = 39**	**Controls non febrile** **N = 35**	P* 2 × 2^a^ **2 × 2^b^**
Box	Worse	11.6 (5)	16.3 (7)	11.4 (4)	0.757 0.455
Cube	Worse	14.0 (6)	16.3 (7)	31.4 (11)	1.000 0.186
Ring	Worse	11.6 (5)	11.6 (5)	11.4 (4)	1.000 0.285
Coin	Worse	4.7 (2)	2.3 (1)	2.9 (1)	1.000 0.178
Behaviour	Worse	11.6 (5)	14.0 (6)	31.4 (11)	1.000 **< 0.001**
Tone	Decreased	41.9 (18)	40.5 (17)	8.8 (3)	1.000 **0.026**

		Day 28 - Day 0			
Box	Worse	5.1 (2)	7.7 (3)	9.7 (3)	1.000 0.686
Cube	Worse	15.4 (6)	20.8 (8)	16.1 (5)	0.769 1.000
Ring	Worse	5.1 (2)	7.7 (3)	25.8 (8)	1.000 **0.009**
Coin	Worse	5.1 (2)	5.1 (2)	9.7 (3)	1.000 0.403
Behaviour	Worse	15.4 (6)	10.3 (4)	45.2 (14)	0.737 **0.001**
Tone	Decreased	51.3 (20)	44.7 (17)	19.4 (6)	0.650 **0.009**

*P value comparison of proportions ^a ^of 2 × 2 for AS7 *vs *MAS7, ^b ^2 × 2 tables for malaria treatment pooled *vs *controls

### Analysis of performance on day 9, 10 and 14 compared to day 7

The proportion of children with worse scores for items of hand co-ordination on days 9, 10 and 14 compared to day 7 items, was not significantly different between mefloquine-and artesunate-treated children and those treated with artesunate alone (Table 4). Likewise behaviour scores were not significantly worse and tone scores did not decrease significantly once mefloquine treatment was given to the children (Table 4).

**Table 4:** Proportion of children with worse performance on day 9, 10 and 14 compared to day 7, in children with *P. falciparum *treated with AS7 or MAS7.

		**Day 9 - Day 7**		
**Neurological Test Component**	**Result compared to previous test**	**AS7 N = 39**	**MAS7 N = 39**	P*
Box	Worse	17.9 (7)	20.5 (8)	1.000
Cube	Worse	15.4 (6)	28.2 (11)	0.272
Ring	Worse	12.8 (5)	20.5 (8)	0.545
Coin	Worse	12.8 (5)	12.8 (5)	1.000
Behaviour	Worse	33.3 (13)	30.8 (12)	1.000
Tone	Decreased	46.2 (18)	53.8 (21^)	0.651

		**Day 10 - Day 7**		
		AS7 N = 39	MAS7 N = 39	P*
Box	Worse	12.8 (5)	15.4 (6)	1.000
Cube	Worse	23.1 (9)	33.3 (13)	0.457
Ring	Worse	5.1 (2)	20.5 (8)	0.087
Coin	Worse	7.7 (3)	12.8 (5)	0.711
Behaviour	Worse	25.6 (10)	30.8 (12)	0.802
Tone	Decreased	38.5 (15)	50 (19^)	0.363

		**Day 14 - Day 7**		
		AS7 N = 38	MAS7 N = 38	P*
Box	Worse	15.8 (6)	21.1 (8)	0.768
Cube	Worse	23.7 (9)	28.9 (11)	0.795
Ring	Worse	15.8 (6)	2.6 (1)	0.108
Coin	Worse	10.5 (4)	7.9 (3)	1.000
Behaviour	Worse	26.3 (10)	28.9 (11)	1.000
Tone	Decreased	34.2 (13)	56.8 (21^)	0.084

*comparison of 2 × 2 for AS7 *vs *MAS7

^Tone 1 less than N for MAS7 on each comparison

### Comparison of timed coin box item at each day of examination

For this item children were required to put 6 or 12 coins into the coin box as fast as they could. Approximately half the children were unable to score on this item (Figure 1). Improvement on this item was reflected by a reduced time (seconds) to complete the task. The median time for each day of testing was summarized and paired data was compared for the specified days (Table 5). The mean age of children who managed to score on this item was significantly higher than those who did not complete this task: 6 coins; 3.4 ± 0.9 *vs *1.7 ± 1.0 yrs and 12 coins; 3.4 ± 0.9 *vs *1.8 ± 1.1 yrs, P < 0.001 for both. Nevertheless there was no trend for significantly worse times at day 7 compared to day 0 when an effect of artesunate may be noticed, nor at day 9, 10 and 14 compared to day 7, when an effect of mefloquine may be noticed. Although there are some significantly faster times in the artesunate-mefloquine group compared to artesunate alone group on day 7 and 9 and day 7 and 10, a practice effect cannot be ruled out.

**Table 5:** Paired comparison of the time (median [min-max] seconds) to put coins into the coin box at each specified day

**Days of comparison**	**Time**	**N pairs**	**Controls (well, no fever)**		
			**Earlier day**	**Later day**	P*
0-7	6 coins	18	13.8 [11-32]	13.8 [11-27.5]	0.722
	12 coins	18	31.5 [23-94]	30 [23.5-55.5]	0.554
0-28	6 coins	15	14 [11-32]	14 [9-18]	0.172
	12 coins	14	32 [25-94]	29 [22-50]	0.158
7-9	6 coins	n.a	n.a	n.a	n.a
	12 coins	n.a	n.a	n.a	n.a
7-10	6 coins	n.a	n.a	n.a	n.a
	12 coins	n.a	n.a	n.a	n.a
7-14	6 coins	19	15.0 [10-48]	14.8 [11-39.5]	0.157
	12 coins	17	30.5 [23.5-55.5]	31.5 [23.5-69.0]	0.820
7-28	6 coins	17	14.5 [10.5-35.5]	13.5 [8.5-23.5]	0.055
	12 coins	16	29.5 [23.5-76]	29 [22-83]	0.510

			**Malaria - AS7**		
0-7	6 coins	12	12.3 [10-26.5]	13.5 [10-35.5]	0.166
	12 coins	12	27.5 [22-68.5]	26.5 [22-58.5]	0.413
0-28	6 coins	10	13 [10-27]	13 [9-23]	0.673
	12 coins	9	28 [22-69]	28 [20-73]	0.260
7-9	6 coins	15	14.5 [10-35.5]	13.5 [7.5-31]	0.459
	12 coins	14	27.0 [21.0-58.5]	27.5 [20-69]	0.126
7-10	6 coins	16	15.3 [10-35.5]	16.8 [8.5-30.5]	0.529
	12 coins	14	32.0 [21.0-58.5]	33.5 [20-52.5]	0.700
7-14	6 coins	14	14.3 [10.5-35.5]	17.3 [8.0-28.5]	0.201
	12 coins	12	25.8 [21.0-58.5]	29.0 [19-78.0]	0.480
7-28	6 coins	13	14 [10.5-35.5]	14 [8.5-22.5]	0.059
	12 coins	11	25.0 [21.0-56.0]	28.0 [20-55.0]	0.722

			**Malaria - MAS7**		
0-7	6 coins	18	15 [10-23]	14.5 [7-30.5]	0.604
	12 coins	18	32.3 [21-47.5]	29.8 [20-51]	0.066
0-28	6 coins	16	15 [10-23]	14 [6-26]	0.277
	12 coins	14	33 [21-48]	30 [20-62]	0.220
7-9	6 coins	20	15.8 [7-34.5]	13.8 [8-31]	0.067
	12 coins	19	33 [20-65.5]	30 [18-50]	**0.040**
7-10	6 coins	20	15.5 [7-34.5]	13.3 [8.5-26]	**0.030**
	12 coins	18	30.5 [20-52.5]	30.8 [18-67.5]	0.924
7-14	6 coins	19	15.5 [7-34.5]	14.5 [8.5-33.0]	0.573
	12 coins	17	31.0 [22.5-52.5]	31.0 [20.0-70]	0.135
7-28	6 coins	20	15.5 [7-34.5]	14.3 [6-28.5]	**0.025**
	12 coins	18	30.5 [20.-61.5]	30.0 [20-61.5]	0.316

*P = Wilcoxon Signed Ranks Test for paired samples at specified days; N = number of pairs analysed

## Discussion

This study represents the first attempt to focus specifically on the occurrence of CNS effects in young children following a seven-day treatment with either artesunate monotherapy or artesunate-mefloquine combination therapy for acute uncomplicated falciparum malaria. Performance on items testing co-ordination, behaviour and tone was not significantly worsened by either treatment. The comparison of proportions of worse scores from day 28 to day 0 suggests there are no long-lasting effects of malaria treated either with artesunate monotherapy or mefloquine-artesunate combination therapy.

Although the numbers of children able to perform the timed test of 6 and 12 coins into the slot in the coin box were limited, the data is useful as it clearly demonstrates no severe nor frequent adverse effect of artesunate or mefloquine on a test requiring concentration, co-ordination and a functional level of tone. Indeed these results are in keeping with the few detailed psychomotor studies in adults [13-17] where mefloquine (and artesunate) do not appear to be detrimental to coordination and behavior.

The tone and behaviour tasks were based on manipulation and observation of the child and were therefore consistently completed. However there were difficulties with interpreting tone and behaviour scores since the scores for both were significantly influenced by the presence of fever and vomiting. As well tone was negatively correlated with behavior. There was a significant improvement in behaviour by day 7 and a further gain by day 28. Similarly, there was a significant reduction in tone with treatment by day 7 and further still by day 28. Most of the improvement was present at day 7 presumably due to the resolution of fever and vomiting and clearance of malaria parasites.

Assessing the neurological status of young children who are acutely unwell with malaria is extremely difficult [38,39]. *Plasmodium falciparum *infection is likely to negatively influence CNS function [40-43], reflected in the observation that some elements of the assessment were completed by only 30% of children on day 0. Those who were the most unwell were unable to cooperate with the tests, and conversely, those who were able to cooperate were the least unwell and in this study were able to score as well as controls. It could be argued that the ability to participate in the test represents an equally useful evaluation of neurological status in children with malaria as does the actual score achieved when testing is possible. *P. falciparum *infection is associated with fever and the children with fever were less likely to have a valid test score in this study. Yet *P. falciparum per se *affects neurological function [40-43], and this is reflected in the observations that in young, unwell children paediatricians typically examine for fever and hypotonia [44] but the tone scores in *P. falciparum *affected children were highest on admission: with fever being associated with higher tone scores which became lower following treatment. Treating falciparum malaria with either regime significantly improved the rates of valid test scores, representing an improvement in neurological function that may overwhelm any possible subtle neurological side-effect of artesunate or mefloquine.

It must be conceded that the ideal test conditions did not apply in this setting. Preferably one would know the baseline abilities per item of children tested without the presence of illness. To establish the baseline level children would need repeated testing at different ages to account for normal developmental growth. Alternatively, by studying a larger number of children, age norms could be established or by inclusion of febrile *P. vivax *and febrile non-malaria children, *P. falciparum *effects could be differentiated from fever effects. Sophisticated field equipment, such as audiometry testing is another method that might be used but even this can be affected by malaria fever [28]. However, under difficult circumstances these tests have provided preliminary information on the safety of these drugs in young children. The neurological tests proved sufficiently sensitive to detect changes in performance in these children over time and paired data analysis allowed a powerful statistical test to determine recovery. A greater sample size may have allowed more subtle differences to be detected. A subtle effect of mefloquine may be more readily detected in future studies of mefloquine where "well" infants receive the drug as intermittent preventive treatment. Another limitation of the study was the length of follow up and a further examination at day 42 or day 63 would have been useful to confirm if the rising trend for increased valid test scores conformed with the control children.

In the past 15 years, 8,172 children under five years have been treated for falciparum malaria most of whom received the combination of artesunate and mefloquine and approximately 564 have been followed up weekly (6-9 weeks) for side effects in chemotherapy studies at Shoklo Malaria Research Unit. Very few CNS related adverse events have been observed with this regimen when the ground rules are adhered to. They include withholding mefloquine when the malaria is cerebral or severe; when the patient is epileptic; when there is a history of psychiatric problems, convulsions or previous problems on mefloquine; when mefloquine has been used for treatment in the past two months; or when the patient weighs less than 5 kg. As shown in this small study, mefloquine clearly reduces the risk of developing *P. vivax *during follow-up and the mefloquine-artesunate combination was not associated with worse performance on neurological testing. Given these findings, and the potential long-term complications of poorly-treated malaria infection in children [39,45], mefloquine-artesunate combination therapy should continue to be administered to children with acute uncomplicated *falciparum *malaria.

## Conclusion

Assessment of neurologic function in acutely unwell children with *P. falciparum *is difficult and affected by fever and vomiting. Treatment results in a significant improvement in the ability of children to participate in neurological testing and no worsening of performance on those tests. Neither artesunate nor mefloquine resulted in significant impairment of behavior or motor function in very young Karen children when compared with non-febrile controls.

## Competing interests

The authors declare that they have no competing interests.

## Authors' contributions

CL and RM were responsible for clinical care of the patients. LD, CL, RA and EPH participated in the testing of children and helped draft the manuscript. KLT, JV, MTA and RM participated in the analysis of data and drafting of the manuscript. LD participated in the design of the study. MTA, RM and FN performed the statistical analysis. FN and CL conceived of the study, and participated in its design and coordination and PS helped to draft the manuscript. All authors read and approved the final manuscript.

## Author Information

LD is an Associate Professor at the Hammersmith Hospital in Paediatric Neonatology with a broad experience of working with young children in resource poor settings.
